# Physical Therapy Use and Associated Factors in Adults with and without Osteoarthritis—An Analysis of the Population-Based German Health Update Study

**DOI:** 10.3390/healthcare9111544

**Published:** 2021-11-12

**Authors:** Kim Elisa Sussmann, Hannes Jacobs, Falk Hoffmann

**Affiliations:** Department of Health Services Research, Carl von Ossietzky University Oldenburg, 26129 Oldenburg, Germany; hannes.jacobs@uni-oldenburg.de (H.J.); falk.hoffmann@uni-oldenburg.de (F.H.)

**Keywords:** osteoarthritis, physical therapy, associated factors, health services

## Abstract

Background: Physical therapy (PT) is recommended as first-line management for osteoarthritis (OA). The purpose of this study was to assess the PT use among adults with OA and those without (Non-OA) and subsequently identify associated factors among these populations. Methods: This cross-sectional study obtained national data from the population-based German Health Update (GEDA2014/2015-EHIS) study containing 24,016 participants aged 18 years and older. Analyses were stratified by sex, age, socioeconomic status (SES), residence, smoking behavior, body mass index, pain and general health. Multivariate regression analysis was conducted to evaluate factors associated with PT use within the past 12 months. Results: PT was used more frequently in the OA population compared with the Non-OA population (35.8% vs. 18.7%). In both populations, women, participants with high SES, residence in Eastern Germany, severe pain, poor general health and non-smokers received PT more frequently. Multivariate analysis confirmed these findings, in addition to people aged 80 years and older. The influence of SES was higher among OA participants. Conclusion: The underutilization of PT in OA patients (35.8%) was particularly evident among males, people with a low SES and those being older than 60 years, which aids to develop strategies increasing PT use towards guideline-oriented OA management.

## 1. Introduction

Osteoarthritis (OA) is the most common disease of the joint in adults in industrialized countries [1]. It is one of the leading causes of disability worldwide, resulting in a great financial burden for health care systems [2,3,4]. Age, obesity and female sex are three of the main risk factors for OA development [5]. Due to the demographic change and an expected rise in obesity, OA will become even more prevalent in the future [6].

The main complaints of patients suffering from OA are progressive limitations in mobility and constant pain, both heavily impacting the patient’s quality of life [1]. One essential cornerstone in the treatment of OA is physical therapy (PT) which aims to restore and maintain joint function, reduce pain and aid in weight loss [7,8,9,10]. In combination with education and analgesics, German and international guidelines highlight PT as the first-line option for OA management, forming the most effective intervention in postponing surgery [1,5,11].

However, a lack of adequate implementation of the OA guidelines has resulted in an underutilization of PT services [1,10,12]. In the year prior to their surgery, only 49% of OA patients received PT in Germany, while this proportion was even lower among other Western countries, such as the US, Australia and Taiwan [10,13,14,15].

Despite an abundance of research conducted on the factors influencing PT use, certain groups of individuals within the OA population remain disproportionately undersupplied by PT. Conversely, women, people with a bachelor’s degree or higher and individuals with a longer symptom duration are more likely to receive PT [15,16,17]. Among the general population, PT was also more likely to be used by females, individuals with a higher educational status or private health insurance, non-smokers, individuals receiving social support and migrants. Physical therapist availability also played a role in the likelihood of receiving PT [18,19]. Despite consensus on the association of the aforementioned factors, the influence of several other factors remains contradictory. Yeh et al., for example, determined age as a predictor for PT use among OA individuals, whereas Iversen et al. found no association between age and PT utilization in this patient group [15,16]. Among the general population, the association between age and PT utilization is also contradictory [18,19,20,21,22]. To date, no study compared PT use among OA patients versus the total population using the same methodology making it possible to comprehensively compare PT use among the OA population with the total population.

Hence, the aim of the present study is to assess and compare the frequency of PT utilization among OA patients and individuals from the general population without OA in Germany, and to evaluate factors associated with PT use among both groups.

## 2. Materials and Methods

### 2.1. Study Design

Data were obtained from the German Health Update (GEDA2014/2015-EHIS) study which was carried out as an adjunct of the European Health Interview Survey (EHIS) [23,24]. The purpose of EHIS is to gain comparable cross-sectional health data across European countries in order to monitor trends. GEDA2014/2015-EHIS was performed by the Robert Koch-Institute (RKI). A two-stage cluster sampling procedure was applied in this population-based survey to adequately represent the German adult population. During the first stage, a random selection of 301 primary sampling units (PSUs) was performed in order to adequately represent all regions of Germany (e.g., federal states with a small number of inhabitants were oversampled). The second stage involved a random sample of participants with a permanent residence in a German household within each PSU taken from local registration offices. The aim was to gather a sample size of *n* = 67 per PSU drawn with stratification according to age [25].

The survey was conducted from November 2014 until July 2015 using a mixed-mode-design compromising either an online or a paper-based self-administered questionnaire [25]. Both the online and paper-based version included the same questionnaire developed by EHIS plus a second survey which evaluated nationally relevant topics. An initial invitation with the link to the online survey was sent. If no answer was received, up to two reminders, each containing the paper-based form, followed. To avoid seasonal variations, the study population was randomly divided into two subgroups: the first group was contacted during fall and winter 2014 and the second group during spring and summer 2015 [26].

### 2.2. Variables and Instruments

The survey assessed a wide range of data on sociodemographics, mental and physical health status, utilization of health care services, working and social environment, as well as health determinants of the participants. The presence of OA was assessed by the question ‘Did you suffer from any of the following diseases or complaints within the past 12 months? Were you diagnosed with any of the following diseases or complaints?’ whereby several diseases were listed including OA. Whether PT was used was evaluated by the question ‘Were you at a physical therapist for consultation, examination or therapy within the past 12 months?’.

The following eight measures were selected as being independent variables of particular interest. As sociodemographic variables, sex (male, female), age (categorized into 18–39, 40–49, 50–59, 60–69, 70–79 and 80 + years), region of residence (Eastern and Western Germany) and socioeconomic status (SES) were included. Using the index developed by the RKI which included information on education, income and profession, SES was divided into three categories (high, middle and low) [27]. Furthermore, the variables smoking behavior (yes, no), body mass index (BMI) categorized into recommended body weight (<25 kg/m^2^), overweight (between 25 and 30 kg/m^2^) and obesity (above 30 kg/m^2^) and general health status (good, fair, poor) were assessed. Lastly, physical pain was evaluated by asking ‘How intense was your pain within the last 4 weeks?’ classified into no pain, mild to moderate and severe.

### 2.3. Statistical Analysis

Only participants who completed the abovementioned question regarding disease diagnosis were included. Besides the total population including all participants, this resulted in two subpopulations: participants being diagnosed with OA (OA population) and participants not having the disease (Non-OA population). Due to missing values, the denominator in the stratified analyses might vary.

Descriptive analysis including percentages and 95% confidence intervals (CI) was used to determine baseline characteristics stratified according to OA and Non-OA population. Thereafter, PT utilization in the total sample and among the two subpopulations stratified by the covariates sex, age, SES, residential region, smoking behavior, BMI, pain and general health status was descriptively assessed. Multivariate regression analysis was used to identify factors associated with PT utilization among OA, Non-OA and total population. In these models, the covariates sex, age, SES, residential region, smoking behavior, BMI, pain, general health status and, only in the last model (total population), OA were included.

Data analyses were conducted using the complex sampling function in IBM SPSS Statistics for Windows, version 26 (IBM corp., Armonk, NY, USA). Unweighted counts were provided for each analysis. Proportions and odd ratios (ORs) were weighted according to sex, age and region in order to represent German population structure.

## 3. Results

### 3.1. Characteristics of Study Population

The initial GEDA2014/2015-EHIS included a total of 24,016 respondents aged 18 years and above (response: 26.9%), of which 1263 did not answer the question regarding OA disease diagnosis. Of the 22,753 respondents, 17.9% were diagnosed with OA. When comparing respondents with and without OA, several differences became apparent (Table 1). The OA population contained more women (62.0% vs. 48.6%). This population was also older with 88.1% of participants aged above 50 (vs. 41.5%), had a higher proportion of individuals with a BMI above 25 kg/m^2^ (69.8% vs. 50.3%) and estimated their general health status more often as poor (14.0% vs. 3.5%). Furthermore, the OA group showed a higher proportion of participants experiencing pain (mild–moderate: 65.3% vs. 50.9%; severe: 24.0% vs. 7.2%). SES, residence and smoking behavior were similarly distributed in both populations.

### 3.2. Frequency of PT Utilization

The frequency of PT utilization within the past 12 months among the total population was 21.8% (Table 2). It appeared that women use PT more frequently than men (25.7% vs. 17.7%). Regarding SES, most PT was received by participants with a high SES (23.3%). Furthermore, people living in Eastern Germany used PT more often than the Western German population (26.4% vs. 20.6%). Non-smokers reported a higher use of PT in comparison to smokers (22.8% vs. 18.6%). PT frequency increased with higher severity of pain, as well as with worsening of general health status. No significant difference in PT frequency was found between the BMI categories or between age groups for participants older than 40 years.

Comparing the subpopulations OA and Non-OA, the OA participants used PT significantly more frequently (35.8% vs. 18.7%). This was also seen for almost all covariates (Table 2). Similar trends among both populations were shown, yet frequencies were higher among the OA group. In general, the findings of PT utilization among the OA and Non-OA population were the same as in the total sample; women, participants having a high SES, those having an Eastern residence, non-smokers, participants having severe pain and those with a poor general health appeared to use PT more frequently. The influence of SES on PT use was higher among OA participants compared to the Non-OA participants. Regarding age, trends of PT frequency differed between OA and Non-OA participants. PT use among the OA group steadily decreased with increasing age, starting at 42.6% in 50-year-olds and declining to 23.1% in those being over 80. Within the Non-OA group, 50 to 59-year-olds received most PT, while below and above this age, the frequency of PT declined, respectively. This decline was less rapid than in the OA group. There was no statistically significant difference in PT use between the OA and Non-OA population in the subgroup of participants having a poor general health.

### 3.3. Associated Factors for PT Utilization among Total Population

Most of the differences regarding PT use that were identified in the stratified analyses among the total population were confirmed in the multivariate analysis (Table 3). These variables associated with PT use were female sex, a medium to high SES, a residence in Eastern Germany, being a non-smoker, a BMI below 30 kg/m^2^, presence of pain and a fair to poor general health status. The presence of OA was also associated with PT utilization (OR: 1.71). Although not seen in the stratified analyses, an age of 40–59 years was identified as an additional associated factor (OR: 1.22) and participants aged above 80 years were less likely to use PT. Severe pain had by far the highest impact on PT utilization (OR: 3.18).

### 3.4. Associated Factors for PT Utilization: OA vs. Non-OA Population

Factors that were associated with utilization of PT, as well as their influence, were largely comparable between the total and the Non-OA population (Table 3). The influence of SES, however, was slightly lower in the Non-OA group (e.g., ORs: 1.62 vs. 1.86 for high SES) and the impact of pain was higher (ORs: 3.52 vs. 3.18 for severe pain).

In both the Non-OA and OA group, use of PT was associated with female sex, medium and high SES, Eastern residence, non-smoking, presence of pain and a fair to poor general health status (Table 3). BMI was not associated with PT use in the OA population. A large difference between the two populations was found with respect to age; above the age of 60, PT utilization became gradually less with increasing age in the OA population. OA patients receiving PT was highest in the group of 18–39 years. Conversely, OA participants aged above 80 years were least likely to use PT (OR: 0.25). Among the Non-OA population, PT was most likely to be used by individuals aged 40–59 years. Similar to the OA group, participants above the age of 80 were significantly least likely to use PT (OR: 0.63). SES had a higher influence on using PT among the OA population compared to the Non-OA population. Pain, on the other hand, had less of an influence on the OA population (e.g., ORs: 2.14 vs. 3.52 for severe pain). These findings of PT use using multivariate analyses largely confirm the results of the stratified analysis in both populations.

## 4. Discussion

In a large population-based study, PT utilization was almost twice as high in patients with OA (35.8%) in comparison to individuals without OA (18.7%). In both groups, participants with a female sex, a medium to high SES, an Eastern German residence, non-smokers, pain and a fair to poor general health status were more likely to use PT, with some variations in terms of influences. The influence of SES was higher in patients with OA, whereas severe pain had a higher influence in those without OA. Regarding age, a linear decline in the likelihood of receiving PT was seen from the age of 60 years among OA patients whereas this decline was not observed until the age of 80 years in individuals without OA.

Overall, PT utilization in patients with OA was low, which is in line with German literature. In the research conducted by Postler et al., whereby they assessed OA treatment among Germans aged above 60 years, the prevalence was 43% [28]. Similarly, a second German study analyzing PT use among OA patients 12 months prior to surgery reported that 49% of patients received PT [13]. One possible explanation for the lower PT frequency observed in the current paper could be the exclusion of people living in e.g., nursing homes, who have more comorbidities or other further existing disabilities that could also be indications for PT. That said, the underutilization of PT is an international issue; in Australia only 39% of OA patients received a referral for PT [14]. Possible reasons for the lack of PT in OA patients seem to be diverse. Literature states that financial barriers, the lack of health-insurance reimbursements and physician’s characteristics, such as inadequate knowledge and negative medical attitude towards the effect of PT, may impact the use of PT [10,13,15,19,29]. This should be addressed in future research.

In both populations, PT was more likely to be used by women, substantiating findings in German and international literature. Commonly, women use health care services more frequently than men [17,18]. The higher prevalence of OA in women, the increased disease severity and a longer life expectancy add to the increased likelihood [16,17]. An additional explanation is that men tend to use fast-relieving pain methods more often than women [16].

The present findings show a clear influence of SES on PT use, not only in the OA population, but also in the total population. The impact of a higher SES on PT use among patients with OA is in line with previous research [15,16,30]. However, in the total population, literature remains controversial and no conclusive evidence has been reported. Freburger et al., assessing PT utilization in elderly Americans, also determined an association between SES and PT use [19]. This association was also identified among patients suffering from musculoskeletal diseases such as lower back pain or neck pain [20,21,31]. For other diseases, however, no association between PT use and SES was found. Jacobs et al. reported in their cross-sectional study that income had no association with PT utilization in German patients with rheumatic disease [32]. Moreover, with regards to spinal cord injury, no association was found between education and receiving PT [33]. This could be due to the fact that the use and effectiveness of PT in the aforementioned diseases are more commonly known and accepted by physicians who, therefore, tend to prescribe PT more frequently. OA patients, on the contrary, might request a PT prescription from their physicians more actively. This may be due to a lack of knowledge about current OA guidelines or negative attitude of physicians towards the effectiveness of PT on reducing OA complaints, having a high influence on patients [29,34]. OA patients who actively request and understand the value of PT are more likely to have a higher SES, explaining the strong impact of SES on PT use among the OA group in the present study. It should also be noted that the differing instruments and variables in various studies, namely income, education or SES score, may be an additional explanation for the variation in findings. Thus, further investigation is needed in future research.

In both the OA and Non-OA population, a decreased likelihood of PT utilization from the age of 80 years was found, however, trends are quite different. Among OA participants, the likelihood steadily decreased with age, starting at 60 years. This association was similarly found in the Taiwanese study by Yeh et al. assessing PT among OA patients, despite using different age categories, namely younger than 55 years, 56–65 years, 66–75 years and older than 76 years [15]. The association with PT use and age was also demonstrated by Postler et al., using age groups in intervals of 10 years [28]. Lange et al. reported a declining frequency of PT use in OA with increasing age, however, a multivariate analysis was not conducted [13]. In conclusion, it became apparent that an age above 60 years has a negative influence on the PT utilization among people with OA. Possible explanations for the limited use with older age may be that physicians tend to prefer prescribing PT for people of working age and those with earlier stages of diseases. The older people get, the higher the disease severity which in turn means that the added value of PT in increasing mobility decreases. Furthermore, the elderly present with more comorbidities and mobility limitations, making it more difficult for them to access and use PT services [15,20]. Nevertheless, future studies should focus on identifying reasons for disparities in PT use according to age, to be able to provide the same care for all OA patients. Focusing on the Non-OA population, it appears that participants older than 80 years are the least likely to use PT services, which is consistent with the study by Freburger et al. [19]. By contrast, a large population-based study assessing PT utilization in chronic lower back pain found no impact of age on PT use [21]. This was also the case for research on PT utilization among the German population by Rommel et al., although age was used as a continuous variable making it difficult to compare [18]. Differences in inclusion criteria, age ranges and national health care systems may also have an impact on the consistency of the findings. This emphasizes the need for future studies that include large sample sizes in order to assess the effect of age and whether other influencing factors differ by age.

PT use has been shown to increase with pain, which appeared to be highly influential in both populations. Contrary to expectations, the impact of pain was higher in the total population compared with the OA group. This suggests that pain is not the most crucial factor in determining the use of PT services among patients with OA. OA itself as indication for PT may play an additional role for the PT utilization. Furthermore, the effect of PT on relieving the pain may explain the low prevalence of pain reported among OA patients currently receiving PT. Unfortunately, there is a paucity of literature assessing the influence of pain on PT use among OA patients. International research among the total population confirms the impact of pain on PT use [31,35]. Clearly, people having pain experience a higher psychological strain and disease burden resulting in a stronger willingness to improve their complaints [36].

### Strength and Limitations

To our knowledge, the present study is one of the first to assess PT use among the OA population and the total population using the same methods and covariates. The large population-based dataset of approximately 24,000 adults without age restriction enabled us to conduct stratified analyses across all age categories, including 80 years and older.

Due to the cross-sectional study design, causal analysis and therefore the effectiveness of PT in reducing OA complaints could not be assessed. Factors such as pain may be influenced by PT use or additional conservative measures not included in the present study such as the use of pain-relief medication. In addition, PT use may be influenced by these conservative measures. All data were based on self-report which increases the risk of recall bias. Data regarding PT utilization were based on one self-reported question that only specified PT use in the past 12 months. Information was therefore not available on the intensity of PT received and the indication for PT. It is possible that among OA patients PT might have been prescribed for other reasons. Furthermore, people living in a non-private German household, such as a nursing home, were excluded. As a result, particularly elderly and less mobile individuals were excluded. The present findings may therefore represent an underestimation of the actual OA occurrence and PT prevalence. Lastly, the low response of around 27% may result in a selection bias. Stratifying for age and sex, Lange et al. determined that a higher response was seen in participants aged 55 to 74 years and in women until the age of 64 [25]. However, these variables were ultimately weighted in the current study to minimize this bias.

## 5. Conclusions

To conclude, PT use among the OA population is low although frequencies are higher than in the total population. The underutilization of PT in the OA population (35.8%) was more apparent in males, individuals with a low SES and individuals older than 60 years. This knowledge enables future research to develop strategies in order to increase PT use among the undersupplied groups optimizing guideline-oriented management for all OA patients equally.

## Figures and Tables

**Table 1:** Baseline characteristics according to OA (*n* = 22,753, unweighted) in % (weighted) and 95% CI.

Characteristics	Category	OA Population (*n* = 3897, Unweighted)			Non-OA Population (*n* = 18,856, Unweighted)		
		%	95% CI	Unweighted Count	%	95% CI	Unweighted Count
Sex (*n* = 22,753)	Male	38.0	36.0–40.0	1482	51.4	50.6–52.3	8790
	Female	62.0	60.0–64.0	2415	48.6	47.7–49.4	10,066
Age, years (*n* = 22,753)	18–39	3.2	2.6–4.0	130	38.6	37.8–39.4	6960
	40–49	8.7	7.7–9.8	359	19.8	19.3–20.5	3937
	50–59	21.7	20.2–23.3	836	18.2	17.7–18.8	3565
	60–69	24.1	22.5–25.7	1015	11.2	10.8–11.7	2208
	70–79	29.7	27.8–31.7	1077	9.2	8.7–9.7	1682
	80+	12.6	11.4–14.0	480	2.9	2.6–3.3	504
SES (*n* = 22,701)	Low	25.8	23.9–27.8	846	18.7	17.7–19.8	2773
	Middle	60.8	58.8–62.7	2279	59.6	58.7–60.6	10,443
	High	13.4	12.2–14.8	762	21.7	20.5–22.9	5598
Residence (*n* = 22,753)	West	81.2	76.4–85.3	3000	79.9	74.9–84.0	13,902
	East	18.8	14.7–23.6	897	20.1	16.0–25.1	4954
Smoking (*n* = 22,701)	Yes	15.2	13.8–16.7	584	25.8	25.0–26.7	4520
	No	84.8	83.3–86.2	3294	74.2	73.3–75.0	14,303
BMI, kg/m^2^ (*n* = 22,540)	<25	30.2	28.4–32.0	1236	49.7	48.6–50.7	9666
	25 ≤ 30	41.3	39.4–43.2	1568	34.5	33.7–35.4	6260
	≥30	28.5	26.9–30.2	1047	15.8	15.0–16.6	2763
Pain (*n* = 22,689)	No	10.7	9.6–12.0	430	42.0	41.1–42.9	7929
	Mild–	65.3	63.4–67.1	2589	50.9	50.0–51.8	9648
	Moderate	24.0	22.3–25.8	865	7.2	6.7–7.7	1228
	Severe						
General Health (*n* = 22,655)	Good	38.1	36.2–40.1	1549	75.5	74.7–76.3	14,512
	Fair	47.9	45.9–50.0	1827	21.0	20.2–21.8	3706
	Poor	14.0	12.6–15.4	487	3.5	3.2–3.9	574

OA = Osteoarthritis; CI = Confidence Interval; SES = Socioeconomic Status; BMI = Body Mass Index; kg = kilogram; m = meter.

**Table 2:** Utilization of physical therapy within OA, Non-OA and total population depending on covariates (*n* = 22,662, unweighted) in % (weighted) and 95% CI.

Characteristics	Category	OA Population (*n* = 3882, Unweighted)		Non-OA Population (*n* = 18,780, Unweighted)		Total Population (*n* = 22,662, Unweighted)	
		%	95% CI	%	95% CI	%	95% CI
Total		35.8	33.8–37.7	18.7	18.0–19.5	21.8	21.0–22.5
Sex	Male	30.7	28.0–33.6	15.6	14.6–16.6	17.7	16.8–18.6
	Female	38.8	36.4–41.4	22.1	21.2–23.1	25.7	24.7–26.7
Age, years	18–39	43.1	33.1–53.6	16.8	15.8–17.9	17.3	16.2–18.4
	40–49	39.3	33.3–45.5	20.8	19.3–22.4	22.4	20.9–24.0
	50–59	42.6	38.8–46.4	21.2	19.7–22.8	25.6	24.1–27.2
	60–69	36.3	32.8–40.0	18.8	17.0–20.8	24.4	22.7–26.2
	70–79	33.9	30.5–37.4	17.9	15.6–20.5	24.6	22.5–26.7
	80 +	23.1	18.7–28.2	16.2	12.5–20.8	19.6	16.6–22.9
SES	Low	28.1	24.7–31.7	16.4	14.8–18.1	19.1	17.7–20.8
	Middle	36.4	33.9–38.9	19.0	18.1–20.0	22.2	21.2–23.2
	High	47.6	43.2–52.0	20.0	18.7–21.4	23.3	21.9–24.7
Residence	West	33.8	31.8–36.0	17.7	16.9–18.5	20.6	19.9–21.4
	East	44.1	39.9–48.3	22.8	21.5–24.1	26.4	24.9–27.9
Smoking	Yes	33.1	28.4–38.1	16.8	15.5–18.1	18.6	17.4–19.9
	No	36.2	34.3–38.3	19.4	18.6–20.3	22.8	21.9–23.7
BMI, kg/m^2^	<25	37.9	34.5–41.4	19.5	18.5–20.5	21.6	20.6–22.6
	25 ≤ 30	34.2	31.5–37.1	18.4	17.2–19.6	21.6	20.5–22.8
	≥ 30	36.4	32.9–40.1	17.7	16.1–19.4	23.0	21.4–24.6
Pain	No	23.6	18.8–29.0	10.2	9.4–11.1	10.9	10.1–11.8
	Mild–Moderate	35.5	33.2–37.9	23.5	22.5–24.6	26.1	25.1–27.1
	Severe	42.2	38.2–46.3	34.6	31.6–37.7	37.8	35.4–40.3
General Health	Good	29.6	26.8–32.6	15.7	14.9–16.4	17.0	16.3–17.8
	Fair	38.5	35.7–41.3	26.5	24.7–28.3	30.5	28.9–32.0
	Poor	43.5	38.4–48.8	39.6	35.1–44.3	41.4	38.1–44.8

OA = Osteoarthritis; CI = Confidence Interval; SES = Socioeconomic Status; BMI = Body Mass Index; kg = kilogram; m = meter.

**Table 3:** Multivariable logistic regression analysis: associated factors for the utilization of physical therapy within OA, Non-OA and total population.

Characteristics	Category	OA Population (*n* = 3764, Unweighted)		Non-OA Population (*n* = 18,455, Unweighted)		Total Population (*n* = 22,219, Unweighted)	
		OR	95% CI	OR	95% CI	OR	95% CI
Osteoarthritis (ref no)	Yes					**1.71**	**1.53–1.92**
Sex (ref men)	Women	**1.55**	**1.30–1.84**	**1.45**	**1.32–1.59**	**1.46**	**1.35–1.57**
Age, years (ref 18–39)	40–49	0.78	0.45–1.35	**1.23**	**1.09–1.38**	**1.22**	**1.09–1.37**
	50–59	0.78	0.49–1.24	**1.23**	**1.07–1.40**	**1.22**	**1.07–1.39**
	60–69	**0.62**	**0.38–0.99**	1.02	0.87–1.19	1.02	0.89–1.17
	70–79	**0.50**	**0.32–0.78**	0.90	0.74–1.10	0.86	0.73–1.01
	80 +	**0.25**	**0.15–0.43**	**0.63**	**0.45–0.89**	**0.49**	**0.39–0.63**
SES (ref low)	Medium	**1.64**	**1.33–2.01**	**1.38**	**1.21–1.58**	**1.46**	**1.30–1.64**
	High	**3.13**	**2.36–4.15**	**1.62**	**1.39–1.88**	**1.86**	**1.62–2.13**
Residence (ref west)	East	**1.55**	**1.28–1.89**	**1.39**	**1.26–1.54**	**1.43**	**1.29–1.58**
Smoking (ref yes)	No	**1.50**	**1.18–1.93**	**1.31**	**1.16–1.46**	**1.32**	**1.19–1.47**
BMI, kg/m^2^ (ref BMI ≥ 30)	<25	1.24	1.00–1.56	**1.37**	**1.20–1.57**	**1.32**	**1.18–1.48**
	25 ≤ 30	1.11	0.89–1.37	**1.30**	**1.12–1.50**	**1.23**	**1.09–1.39**
Pain (ref no)	Mild–Moderate	**1.66**	**1.22–2.26**	**2.42**	**2.16–2.70**	**2.35**	**2.12–2.60**
	Severe	**2.14**	**1.49–3.07**	**3.52**	**2.93–4.22**	**3.18**	**2.72–3.73**
General Health (ref good)	Fair	**1.70**	**1.40–2.07**	**1.67**	**1.48–1.87**	**1.69**	**1.53–1.87**
	Poor	**2.26**	**1.63–3.15**	**2.80**	**2.19–3.59**	**2.51**	**2.05–3.06**

OA = Osteoarthritis; OR = Odds Ratio; CI = Confidence Interval; ref = Reference Group; SES = Socioeconomic Status; BMI = Body Mass Index; kg = kilogram; m = meter. Boldface indicates significant results (*p* < 0.05).

## Data Availability

The dataset analyzed during the current study is available as a public use file and can be requested upon application from the Research Data Centre of the Robert Koch-Institute. [https://www.rki.de/EN/Content/Health_Monitoring/Public_Use_Files/application/application_node.html;jsessionid=A8E097F2995AE550E3BEB902737BDE2E.internet072] (accessed on 25 July 2020).
